# Microbiota profile in new-onset pediatric Crohn’s disease: data from a non-Western population

**DOI:** 10.1186/s13099-018-0276-3

**Published:** 2018-11-29

**Authors:** Mohammad I. El Mouzan, Harland S. Winter, Assad A. Assiri, Kirill S. Korolev, Ahmad A. Al Sarkhy, Scot E. Dowd, Mohammad A. Al Mofarreh, Rajita Menon

**Affiliations:** 1 Pediatric IBD Research Group, Gastroenterology Division, Department of Pediatrics, King Saud University, P. O. Box 2925, Riyadh, 11461 Kingdom of Saudi Arabia; 2 Mass General Hospital for Children, Pediatric IBD Program Boston, Boston, USA; 3 Department of Pediatrics, Gastroenterology Division, Prince Abdullah Bin Khalid Celiac Disease Research Chair, King Saud University, Riyadh, Kingdom of Saudi Arabia; 4 Physics Department and Bioinformatics Program, Boston University, Boston, USA; 5 MRDNA Research, Shallowater, TX USA; 6 Department of Gastroenterology, Al Mofarreh Polyclinic, Riyadh, Kingdom of Saudi Arabia; 7 Physics Department, Boston University, Boston, USA

**Keywords:** Bacteriome, Inflammatory bowel disease, Saudi children

## Abstract

**Background:**

The role of microbiota in Crohn’s disease (CD) is increasingly recognized. However, most of the reports are from Western populations. Considering the possible variation from other populations, the aim of this study was to describe the microbiota profile in children with CD in Saudi Arabia, a non-Western developing country population.

**Results:**

Significantly more abundant genera in children with CD included *Fusobacterium, Peptostreptococcus, Psychrobacter,* and *Acinetobacter;* whereas the most significantly-depleted genera included *Roseburia, Clostridium, Ruminococcus, Ruminoclostridium, Intestinibacter, Mitsuokella, Megasphaera, Streptococcus, Lactobacillus, Turicibacter,* and *Paludibacter.* Alpha diversity was significantly reduced in stool (p = 0.03) but not in mucosa (p = 0.31). Beta diversity showed significant difference in community composition between control and CD samples (p = 0.03).

**Conclusion:**

In this developing country, we found a pattern of microbiota in children with CD similar to Western literature, suggesting a role of recent dietary lifestyle changes in this population on microbiota structure.

## Background

Crohn’s disease (CD) is the most common phenotype of inflammatory bowel disease (IBD). Although the incidence is highest in Western populations, increasing time trend is reported worldwide in adults and children [1–3]. Despite extensive research, the causes of all phenotypes of IBD remain unknown. However, a multifactorial theory is most likely. In a genetically susceptible individual, environmental factors trigger uncontrolled inflammation [4]. Diet and microbiota are the most likely environmental factors, acting as triggers. It was found that there was an increased risk of CD with a high intake of polyunsaturated fatty acids, omega-6 fatty acids, saturated fats, and meat, but there was a decreased risk with a high intake of dietary fiber, omega-3 fatty acids, vegetables, and fruits [5–10]. The role of microbiota in IBD in general, and CD in particular, has been increasingly recognized. Several studies documented reduced diversity of the microbial community and defined associations of certain taxa with CD, suggesting a role of beneficial and harmful microbes [11–14].

Almost all the literature on microbiota in IBD is regarding Western populations, who have well-defined environmental, cultural, and dietary lifestyles, which are different from populations in developing countries. Since IBD is newly recognized in these populations, these are commonly referred to as “new populations” with IBD. The study of the characteristics of IBD in these populations may increase our understanding of this condition.

In previous reports, we have defined the incidence and clinical profile of pediatric CD in Saudi Arabia [15, 16]. The objective of this report is to describe the bacterial microbiota profile in a cohort of newly-diagnosed treatment-naïve children in Saudi Arabia, a non-Western developing country population in comparison with other populations reported in relevant literature.

## Results

### Patients characteristics

All children were Saudi nationals (17 with CD and 18 controls). The median (range) age was 15 (7.3–17.8) years for the children with CD and 16.3 (3.9–18.6) years for controls. Gender distribution indicated that 11/17 (65%) of the CD and 12/18 (67%) of the control subjects were males.

Dietary history revealed similar prevalence of breastfeeding in children with CD (13/17; 76%) and controls (13/18; 72%). The quality of family food intake by children estimated by mothers was very good to good in 6/17 (35%) and 9/18 (50%) in children with CD and controls respectively. Daily fast food consumption was 10/17 (58%) and 9/18 (50%), daily sweetened gaseous drink in 11/17 (65%) and 9/18 (50%) in children with CD and controls respectively. Daily fruit consumption was 1/17 (6%) and 2/18 (11%) and vegetable 3/17 (18%) and 10/18 (56%) in children with CD and controls respectively. In summary, compared to controls, children with CD tended to eat less family food, more fast food and sweetened gaseous drinks, but less vegetable and fruit consumption.

The clinical presentation in the 17 children with CD included abdominal pain in 15 (88%), diarrhea in 14 (82%), weight loss in 13 (76%), blood in stools in 8 (47%), and perianal disease in 3 (18%) children. At presentation, laboratory tests revealed anemia in 8 (47%), thrombocytosis in 6 (35%), elevated erythrocyte sedimentation rate (ESR) in 9 (53%), and high C reactive protein (CRP) in 6 (35%) children. At diagnosis, CD locations were ileal (L1) in 4 (24%) and Ileocolonic (L3) in 13 (76%) children while CD behavior was non-constricting non-penetrating (B1) in 13 (75%) and constricting (B2) in 4 (24%) children.

The clinical presentation of the 18 children classified as controls included recurrent abdominal pain in 9 (50%) children, finally diagnosed as functional abdominal pain, diarrhea in 5 (28%) children, finally diagnosed as nonspecific diarrhea, and 4 (22%) children with rectal bleeding, finally diagnosed as juvenile polyps. Hemoglobin, platelets, ESR, and CRP were normal in all controls.

### CD-associated microbiota

**Fig. 1:**
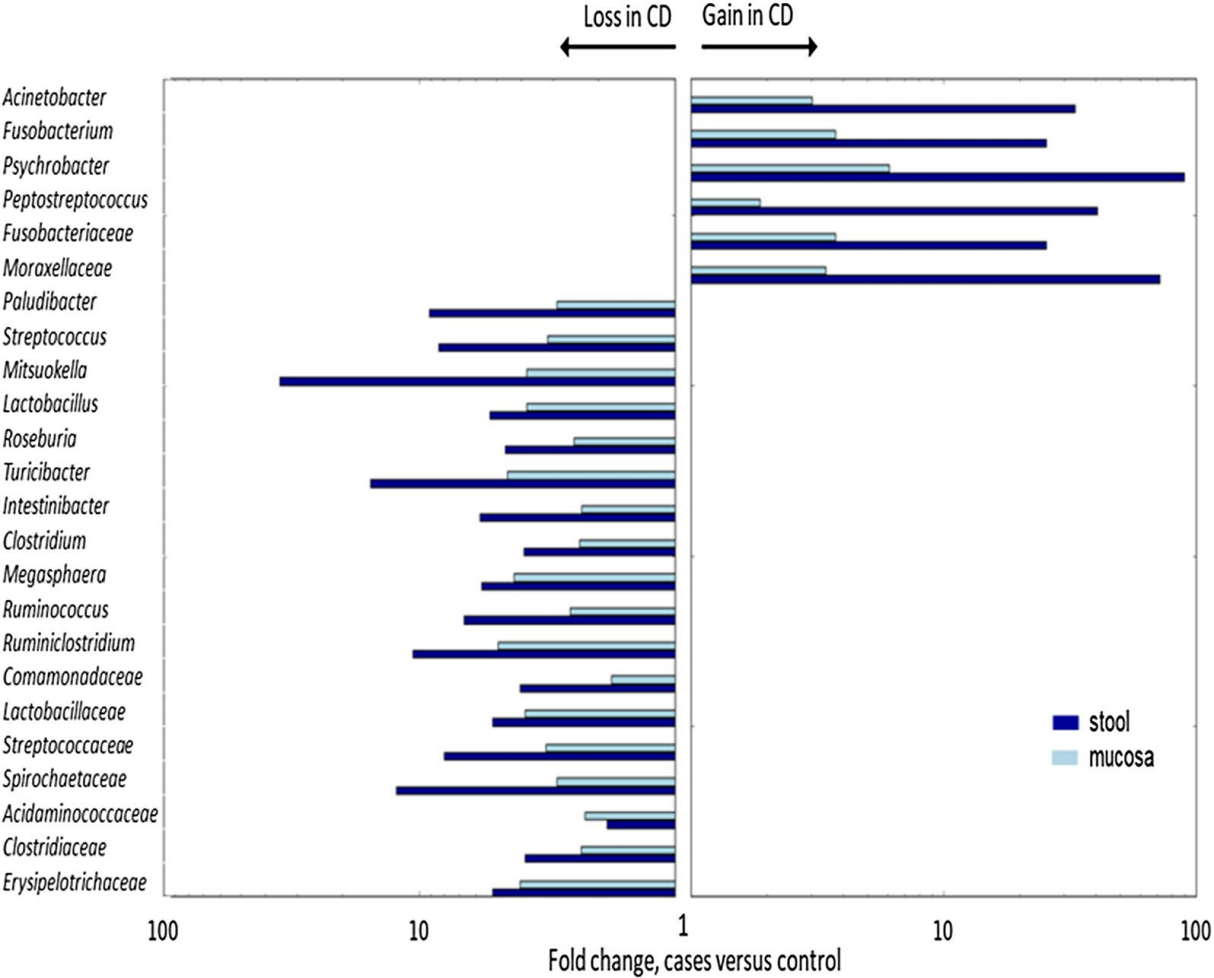
Variation in abundance in CD mucosa and stool. The mean abundance ratio between CD and control of the main phylogenetic taxa at family and genus level. The bars illustrate the direction of change, increase or decrease in CD, indicating gain or loss of taxa respectively. The fold change is the ratio of the mean abundance in CD in relation to controls

**Fig. 2:**
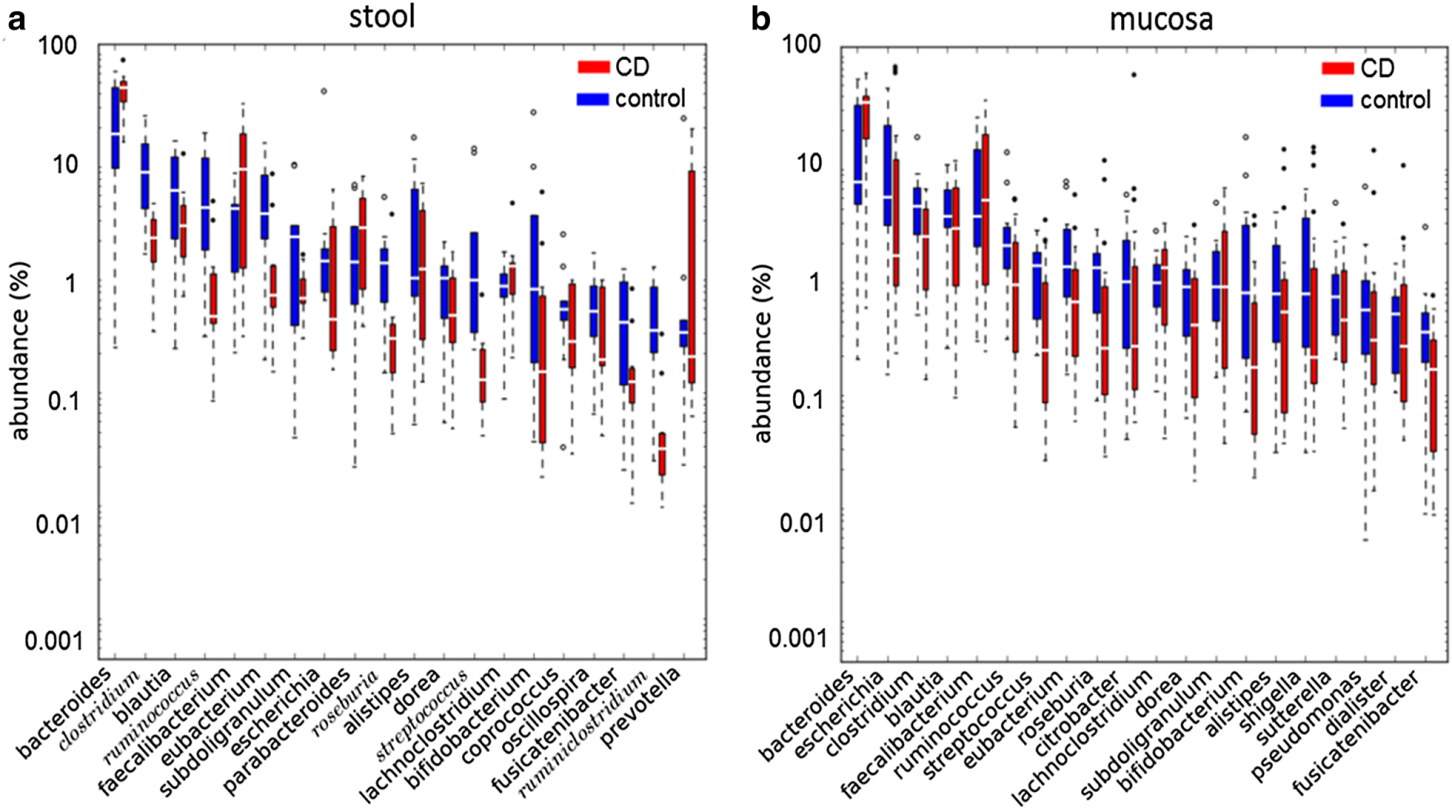
Rank abundance distribution. Abundance of the 20 most abundant genera for **a** stool communities and **b** mucosa communities. The taxa are ordered by median abundance in controls, and their abundance distribution is visualized by a box plot showing the median and the deviant points lying outside the inter-quartile range for each genus. The significant taxa with FDR corrected p value < 0.05 are indicated in italics

The CD-associated taxa for the family, genus, and species phylogenetic levels in stool and mucosa are presented in Tables 1 and 2 respectively. Significantly-abundant taxa in children with CD compared with controls at the genus level included *Fusobacterium, Peptostreptococcus, Psychrobacter,* and *Acinetobacter*, and at the species level, *Fusobacterium nucleatum, Bacteroides clarus,* and *Psychrobacter pulmonis*. The most significantly-depleted genera in children with CD included *Roseburia, Clostridium, Ruminococcus, Ruminoclostridium, Intestinibacter, Mitsuokella, Megasphaera, Streptococcus, Lactobacillus, Turicibacter,* and *Paludibacter*; whereas, significantly-depleted species included *Roseburia inulivorans, Clostridium disporicum, Blaucia ruminococcus* spp*., Eubacterium seraeum, Intestinibacter bartelitii, Eubacterium* spp., *Streptococcus salivarius, Turicibacter* spp., *Bacteroides xylanolyticus, Clostridium perfringens,* and *Bifidobacterium catenulatum*. It is to be noted that no significantly more abundant taxa were found in mucosal samples. By contrast, a large number of taxa were depleted from stool and mucosa samples as detailed in Tables 1 and 2. The direction of change of most taxa (gain or loss) is depicted in Fig. 1 and the rank abundance distribution of the 20 most abundant genera in stool and mucosa samples is illustrated in Fig. 2.

**Table 1:** Fecal bacteria associated with Crohn’s disease

Phylogenetic level	Abundance CD (%)	Abundance control (%)	Ratio	FDR corrected p-value
Order				
Spirochaetales	0.003	0.02	0.11	0.0002
Lactobacillales	0.32	2.42	0.13	0.008
Fusobacteriales	0.32	0.01	23.4	0.01
Erysipelotrichales	0.04	0.23	0.19	0.01
Pseudomonadales	0.33	0.01	22.5	0.01
Rhizobiales	0.02	0.04	0.51	0.02
Actinomycetales	0.01	0.05	0.25	0.03
Family				
Spirochaetaceae	0.003	0.02	0.11	0.00037
Comamonadaceae	0.005	0.02	0.24	0.01
Moraxellaceae	0.23	0.004	58.8	0.01
Fusobacteriaceae	0.32	0.01	23.4	0.01
Erysipelotrichaceae	0.04	0.23	0.19	0.01
Streptococcaceae	0.14	1.23	0.11	0.01
Lactobacillaceae	0.05	0.22	0.22	0.01
Clostridiaceae	1.71	6.7	0.25	0.02
Genus				
*Mitsuokella*	0.002	0.07	0.03	0.0001
*Turicibacter*	0.01	0.19	0.06	0.0018
*Peptostreptococcus*	0.06	0.002	40.7	0.002
*Intestinibacter*	0.04	0.25	0.16	0.002
*Psychrobacter*	0.12	0.001	81.0	0.004
*Ruminiclostridium*	0.03	0.27	0.12	0.011
*Paludibacter*	0.008	0.06	0.13	0.011
*Fusobacterium*	0.32	0.01	23.4	0.011
*Streptococcus*	0.13	1.22	0.11	0.013
*Lactobacillus*	0.05	0.22	0.21	0.013
*Acinetobacter*	0.09	0.003	27.8	0.013
*Megasphaera*	0.01	0.07	0.18	0.013
*Clostridium*	1.67	6.62	0.25	0.02
*Ruminococcus*	0.57	3.72	0.15	0.02
*Roseburia*	0.24	1.04	0.23	0.04
Species				
*Roseburia inulinivorans*	0.02	0.59	0.03	0.00019
*Bacteroides clarus*	0.33	0.01	25.9	0.0002
*Clostridium disporicum*	0.008	0.30	0.03	0.0007
*Turicibacter* spp.	0.01	0.18	0.06	0.0019
*Ruminiclostridium eubacterium siraeum*	0.006	0.14	0.04	0.0019
*Intestinibacter clostridium bartlettii*	0.04	0.25	0.16	0.0019
*Bacteroides xylanolyticus*	0.01	0.35	0.04	0.0019
*Psychrobacter pulmonis*	0.12	0.001	80.9	0.0035
*Ruminococcus* spp.	0.18	1.47	0.12	0.0037
*Streptococcus salivarius*	0.07	1.02	0.07	0.0070
*Ruminococcus flavefaciens*	0.04	0.98	0.04	0.010
*Fusobacterium nucleatum*	0.25	0.01	21.6	0.01
*Eubacterium* spp.	0.07	0.68	0.11	0.02
*Eubacterium hallii*	0.05	0.34	0.15	0.04

*FDR* false discovery rate

**Table 2:** Mucosal bacteria associated with Crohn’s disease

Phylogenetic level	Abundance CD (%)	Abundance control (%)	Ratio	^a^FDR corrected p-value
A. Colonic mucosa				
Family				
Erysipelotrichaceae	0.07	0.26	0.25	0.007
Genus				
* Mitsuokella*	0.009	0.03	0.25	0.001
* Holdemanella*	0.007	0.06	0.13	0.009
* Ruminiclostridium*	0.02	0.12	0.20	0.01
* Turicibacter*	0.02	0.08	0.25	0.02
Species				
* Streptococcus salivarius*	0.1	0.4	0.25	0.05
* Clostridium disporicum*	0.04	0.25	0.15	0.05
B. Ileal mucosa				
Family				
Erysipelotrichaceae	0.03	0.42	0.08	0.02
Lactobacillaceae	0.03	0.56	0.06	0.03
Acidaminococcaceae	0.03	0.26	0.11	0.03
Genus				
* Roseburia*	0.09	1.01	0.09	0.05
* Lactobacillus*	0.03	0.55	0.06	0.05
Species				
* Phascolarctobacterium*	0.03	0.26	0.11	0.05
* Bifidobacterium catenulatum*	0.008	0.17	0.05	0.01
* Clostridium perfringens*	0.02	0.35	0.05	0.03
* Roseburia inulinivorans*	0.04	0.51	0.07	0.03
* Blautia ruminococcus gnavus*	0.08	0.67	0.12	0.04

^a^*FDR* False discovary rate

### Microbiota diversity

**Fig. 3:**
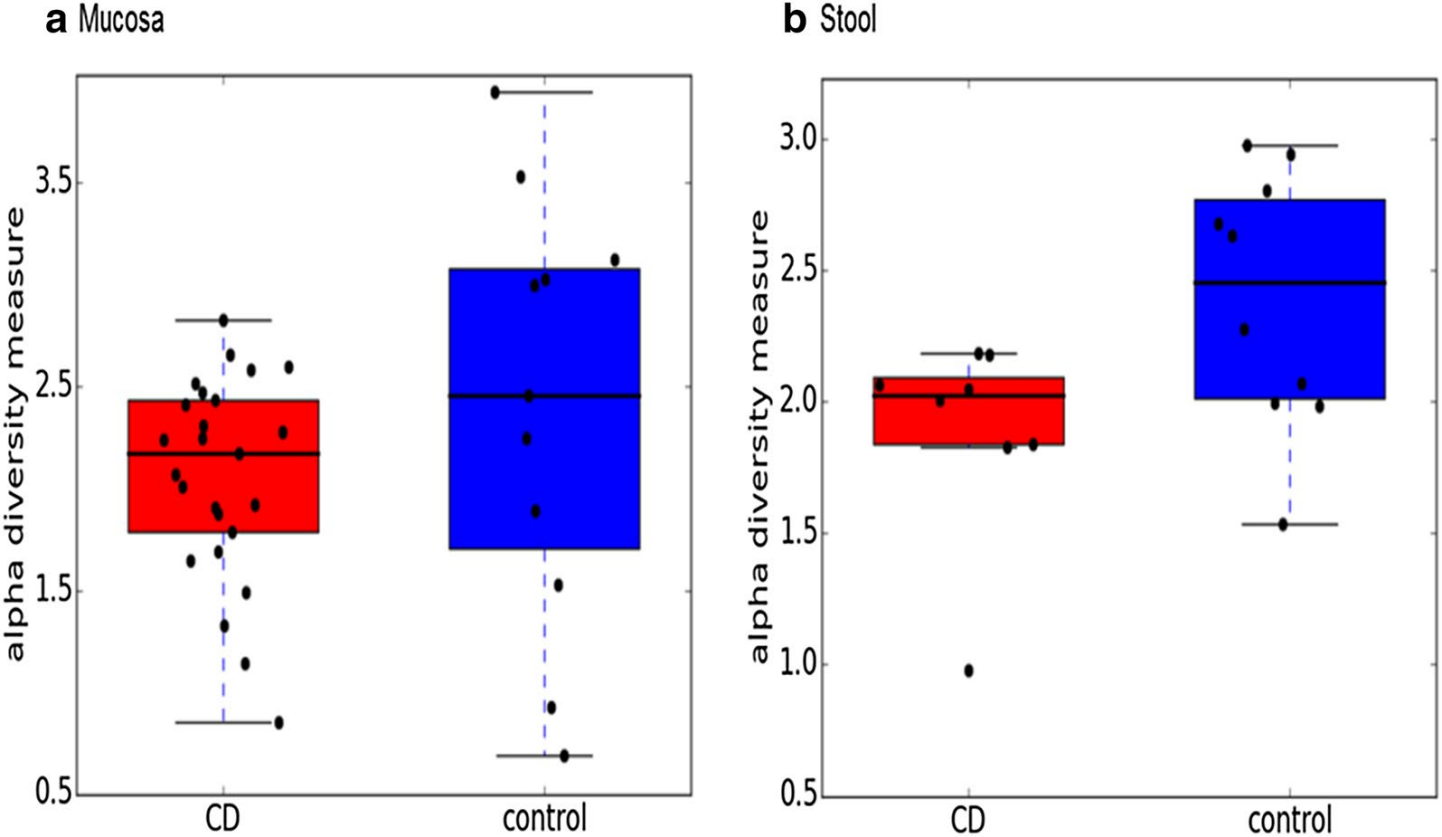
Alpha diversity by Shannon index. Alpha diversity in the stool of children with CD was significantly reduced (p = 0.03); whereas, in CD mucosa te reduction of alpha diversity was not (p = 0.32)

*Alpha diversity*, as measured by the Shannon index, is shown in Fig. 3, indicating significantly-reduced alpha diversity in the stool of children with CD compared to controls (p = 0.03); whereas, the difference in CD mucosa was not significant (p = 0.32).

*Beta diversity* as evaluated by the Bray–Curtis distance indicated a statistically significant community dissimilarity between control and CD samples in stool (p = 0.03).

### Abundance and diversity in inflamed and un inflamed mucosa

Comparison of species abundance between inflamed and uninflamed mucosa of children with CD for the four most different species (*Bacteroides nordii, Escherichia coli, Eisenbergiella tayi, Bacteroides caccae*), indicated no significant difference (p > 0.9). In addition, there was no significant difference in alpha diversity between inflamed and uninflamed mucosa in children with CD (p = 0.31).

## Discussion

The national incidence of pediatric IBD in Saudi Arabia (0.47/10^5^), including CD (0.27/10^5^), has been reported recently, indicating a lower but steady increase in incidence similar to that in the Western literature [15]. In addition, the clinical, laboratory, endoscopic, and histopathologic characteristics have been reported, indicating similar patterns to descriptions from Western countries [16, 17]. In Saudi Arabia, marked socioeconomic improvement which led to improved education, nutrition, and health care, was accompanied by changes in lifestyle. For example, in a larger report on Saudi children with CD, only 10% and 30% consumed fruits daily and twice weekly respectively; whereas about 50% and 30% consumed fast food and sweetened soft drinks daily and twice weekly respectively [18]. This dietary lifestyle pattern indicating less consumption of fruits and high consumption of fast food and sweetened soft drinks is similar to descriptions in the Western literature [19, 20].

IBD in Saudi Arabia evolved from occurring rarely, to a commonly-diagnosed condition with increasing incidence, suggesting a role of recent changes in environment including dietary lifestyle. It is well known that dietary components acting directly or through alteration of intestinal microbiota have a significant role in triggering inflammation [21–24].

This report, to our knowledge, is the first description of microbiota profile in newly-diagnosed treatment-naïve children with CD from a non-Western population. We identified a large number of taxa in the CD fecal and mucosal samples from order to species levels in the phylogenetic tree. However, it should be noted that taxonomic species designations based upon 16 s are tentative assignments and caution is advised in interpretations related to species classification. In view of the variation of the microbiome between newly-diagnosed and treated patients with IBD [25], comparison will be mainly with the few most similar reports of newly-diagnosed and treatment-naïve children with CD.

### CD-associated microbiota

All of the 20 most abundant taxa from the order to the species levels in this study, have been reported in the Western literature [4, 14, 26, 27]. However, the significance of associations of some taxa with CD, contrasts with reports from Western populations. For example, Enterobacteriaceae, reported significantly more abundant in CD [14], was not found significantly associated with CD in our study. Similarly, *Faecalibacterium prausnitzii*, which is reported as significantly-depleted bacteria with possible anti-inflammatory properties [28], was not found to be significantly associated with CD in our samples. Variations in the significance of associations exists between studies even from the same Western populations. For example, at least two reports found *Faecalibacterium prausnitzii* to be more abundant in mucosal samples of children with CD, contrasting with other reports of the protective role of this bacterium in patients with CD [29, 30]. These observations are in line with the well-known variability of microbiota both within and between subjects in the same population. Finally, it is important to note that associations described in this study as well as in the literature, do not imply functional or causal effects. Specifically, it is still unclear whether changes in microbiota in children with CD were the cause or the result of inflammation.

### Microbiota diversity

In this study, alpha diversity was reduced in mucosa and stools of children with CD relative to controls, a finding similar to that in the literature [31]. However, this reduction was statistically significant only in stools (p = 0.03) and not in tissue samples (p = 0.32). Beta diversity in our cohort indicated an overall statistically-significant distance difference between CD and control samples (p = 0.03), a finding similar to reports from Western populations [14].

### Lack of difference between inflamed and uninflamed CD mucosa

In this study, the lack of significant difference in the four most abundant bacterial species and alpha diversity between sites with inflamed and uninflamed mucosa are consistent with most reports and suggest an unlikely role of bacteria in the pathogenesis of the patchy distribution of lesions in CD [32, 33]. However, one report suggested that uninflamed tissue forms an intermediate bacterial population between controls and inflamed tissues [34], and another reported a significant difference in microbial community structure between inflamed and uninflamed mucosal sites, but there was great variation between individuals, suggesting no obvious bacterial signature that was positively associated with the inflamed gut [35]. It appears, therefore, that our findings of no significant difference in the bacterial community between inflamed and uninflamed mucosa in children with CD are consistent with most reports.

In this study from a developing country population, the finding of a microbiota profile similar to that in Western populations was unexpected in view of different culture and lifestyle. However, recent changes to a more westernized dietary lifestyle, affecting microbiota structure, explains at least in part this similarity.

### Study limitations

The most important limitation is the small sample size. However, Crohn’s disease is evolving in this part of the world and the characterization of the microbiota associated with new onset Crohn’s is unique.

## Conclusions

In this developing country, we found a pattern of microbiota in children with CD similar to Western literature, suggesting an effect of recent dietary lifestyle changes on microbiota structure. This report suggests a possible role of dietary lifestyle related to alteration of microbiota and the increasing incidence of CD in the Saudi population.

## Methods

### The study population

In addition to controls, the study population included all children diagnosed with CD according to standard guidelines [36]. The children referred for colonoscopy were enrolled prospectively. Two hospitals participated in the study. These were King Khalid University Hospital, King Saud University (a free-access primary and tertiary care hospital), and Al Mofarreh Polyclinics (a private gastroenterology institution). Demographic information, socioeconomic family status, nutritional history, drug history, history of the present illness, past medical and surgical history, including any medications, physical examination, laboratory, imaging, endoscopic, and histopathological findings were recorded at presentation. Controls were enrolled if they had no evidence of IBD or other causes of inflammation proven by endoscopy and histopathology.

### Sample collection, storage, and processing

In view of the known variations in the microbial community along the gastrointestinal (GI) tract [37–41], mucosal samples were collected from the ileum and different colonic sites to minimize the effects of these variations. Samples were collected from 17 children with CD and 18 controls. A total of 44 tissue samples from the children with CD (8 from the Ileum, 6 from each of the cecum, ascending, transverse, descending, sigmoid colon and rectum) and 14 from controls (6 from the Ileum, 3 transverse colon, 2 sigmoid colon and 3 from the rectum). For logistic reasons, mucosal samples were not taken from the ileum and all colonic segments of each subject. Similarly, not all subjects gave stool samples. A total of 20 stool samples were collected from children with CD (10) and controls (10) before bowel preparation (75%), or from the first stool passed after the start of bowel preparation (25%), to minimize washout effects [41]. All samples were collected in cryovials (no fixatives or stabilizers), immediately placed in ice, transported to the laboratory, and stored at − 80 °C within 5 to 20 min. The average storage duration before analysis was 3 years. At the time of microbiota analysis, all samples were shipped in dry ice by express mail to the USA (MR DNA, Shallowater, TX, USA). The samples were received frozen in about 36 h.

### DNA extraction and sequencing methods

DNA was extracted using the Mobio Powersoil Kit as per the manufacturer’s instructions (MOBIO, Carlsbad, CA, USA). Amplicon sequencing (bTEFAP^®^) was performed at MR DNA (Shallowater, TX, USA) and used for bacterial analysis [42]. The primers 515F GTGCCAGCMGCCGCGGTAA and 806R GGACTACHVGGGTWTCTAAT were used to evaluate the microbial ecology of swabs on the Illumina MiSeq with methods based upon the bTEFAP^®^. A single-step 28-cycle polymerase chain reaction (PCR) with the HotStarTaq Plus Master Mix Kit (Qiagen, Valencia, CA, USA) was employed under the following conditions: 94 °C for 3 min, followed by 28 cycles of 94 °C for 30 s, 53 °C for 40 s, and 72 °C for 1 min; after this, a final elongation step at 72 °C for 5 min was performed. Following PCR, all amplicon products from different samples were mixed in equal concentrations and purified using Agencourt Ampure Beads (Agencourt Bioscience Corporation, MA, USA). Samples were sequenced utilizing Illumina MiSeq Chemistry following the manufacturer’s protocols. The Q25 sequence data derived from the sequencing process was processed using a standard analysis pipeline (http://www.mrdnalab.com; MR DNA, Shallowater, TX, USA). Paired sequences were merged and depleted of barcodes and primers, then short sequences < 150 base pairs (bp) were removed, sequences with ambiguous base calls were removed, and sequences with homopolymer runs exceeding 6 bp were removed. Sequences were then denoised and chimeras were removed. Operational taxonomic units (OTUs) were defined after removal of singleton sequences, clustering at 3% divergence (97% similarity) [43–46]. OTUs were then taxonomically classified using the Nucleotide Basic Local Alignment Search Tool against a 16 s National Center for Biotechnology Information (NCBI)-derived database (http://www.ncbi.nlm.nih.gov, http://rdp.cme.msu.edu), and compiled into each taxonomic level into both ‘counts’ and ‘percentage’ files. Counts files contain the actual number of sequences and percentage files contain the relative (proportional) percentage of sequences within each sample, which map *to the designated taxonomic classification.*

### Statistical analysis

The analysis was performed using Python and R software [47, 48]. To increase statistical power, taxa with low representation in the samples were excluded from the analysis. Specifically, we excluded samples below 1000 reads as well as taxa absent from more than 50% of both CD and control samples. Custom functions implementing the permutation test were written to detect the taxa whose abundances were significantly different between two sample categories, e.g., CD and control or inflamed and uninflamed. When more than one sample was available from the same patient for the analysis, the log-relative abundances from these samples were averaged. We performed all statistical analysis on log-transformed data after adding pseudo counts of 1 read for each taxonomic group.

#### Association analysis

To understand which members of the bacterial microbiota might contribute to CD, we examined the difference in microbial abundance between CD stool and controls, CD mucosa and controls, and inflamed and uninflamed mucosa in CD. First, we compared uninflamed and inflamed mucosa in children with CD and found no significant changes in microbial abundances (p > 0.9). Given this lack of difference, all CD mucosal samples were included in the analysis. Associations were determined based on the difference in the mean log-relative abundance. Statistical significance was assessed via a permutation test (exact Fisher’s test) followed by a correction for multiple hypothesis testing. Specifically, the permutation test yielded raw, uncorrected p-values, which were corrected to q-values following the Benjamini–Hochberg procedure to account for the false discovery rate (FDR) [49]. Although less significantly-associated taxa may be biologically important, we reported only statistically-significant associations when the corrected FDR-corrected p-value was < 0.05.

#### Diversity analysis

Diversity analysis was used to study the richness of taxa and the evenness of habitat composition, as well as the community dissimilarity between samples. *Alpha diversity*, a measure of taxa richness was evaluated by the Shannon index. This measure quantifies the number of taxa and their representative proportions in the habitat; a high alpha diversity indicates that there is a high number of taxa with similar abundance. The difference in diversity between CD mucosa and controls, stool CD and controls, or inflamed and uninflamed mucosa were analyzed. The sample-wise difference in community composition *(Beta diversity)* was quantified by the *Bray*–*Curtis dissimilarity,* which accounts for both patterns of presence-absence of taxa and changes in their relative abundance between samples.

The beta diversity separations were analyzed by the ANOSIM or analysis of (dis)similarity. The ANOSIM statistic compares the mean of ranked dissimilarities between groups to the mean of ranked dissimilarities within groups. The significance of the statistic was determined by an exact permutation test.
